# An Integrative Genotyping and Gene Expression Profiling of the Mutated Human *FAM111B* Gene and Fibrosis‐Associated Pathway in the POIKTMP Syndrome

**DOI:** 10.1111/jcmm.70871

**Published:** 2025-10-06

**Authors:** Nadine Tambwe, Musalula Sinkala, Oluwafemi G. Oluwole, Nonhlanhla P. Khumalo, Afolake Arowolo

**Affiliations:** ^1^ Hair and Skin Research Lab, Division of Dermatology, Department of Medicine University of Cape Town Cape Town South Africa; ^2^ Division of Computational Biology, Department of Integrative Biomedical Sciences, Faculty of Health Sciences University of Cape Town Cape Town South Africa; ^3^ Institute of Infectious Diseases and Molecular Medicine, Faculty of Health Sciences University of Cape Town Cape Town South Africa; ^4^ Biomedical Research Centre, Nuffield Department of Medicine, Centre for Human Genetics University of Oxford Oxford UK; ^5^ Non‐Communicable Diseases Department Institute of Primate Research Nairobi Kenya; ^6^ Biomedical Research and Innovation Platform (BRIP) South African Medical Research Council (SAMRC) Cape Town South Africa

**Keywords:** *FAM111B*, fibrosis, gene enrichment analysis, gene expression, POIKTMP

## Abstract

Poikiloderma with tendon contracture, myopathy and pulmonary fibrosis (POIKTMP) is a rare hereditary disorder caused by mutations in the *FAM111B* gene, characterised by multi‐organ fibrosis, particularly affecting the lungs. This study investigates the molecular mechanisms of fibrosis in POIKTMP through genotyping and gene expression profiling of *FAM111B* and associated fibrotic pathways. Post‐mortem formalin‐fixed paraffin‐embedded (FFPE) tissues from a POIKTMP patient and healthy controls were analysed. Genomic DNA was extracted, confirming the *FAM111B Y621D* mutation via Sanger sequencing. RT‐qPCR and the RT^2^ Profiler PCR Array were used to evaluate fibrosis‐related gene expression in lung and skin tissues. Disease and pathway enrichment analyses were conducted using Metascape, GeneMANIA and Enrichr tools. The *FAM111B Y621D* mutation was validated, and gene expression profiling revealed significant upregulation of fibrotic markers, such as *TGFβ‐3, PDGFA, ITGB1, MMP3, MMP13* and *CCN2* in the lungs, and *COL3A1* and *THBS2* in the skin. Pathway enrichment analysis linked FAM111B to extracellular matrix remodelling, cell adhesion, and cancer. These findings suggest that *FAM111B* mutations drive fibrosis through dysregulated gene networks, highlighting potential therapeutic targets for POIKTMP and related fibrotic diseases. Further research is required to understand *FAM111B*'s role in fibrosis fully.

## 1 Introduction

Fibrosis is the excessive production of fibro‐connective components of extracellular matrices (ECM) in response to inflammation or tissue damage, resulting in abnormal tissue repair [1, 2]. Moreover, fibrosis is a prominent pathological feature of chronic inflammatory diseases such as scleroderma, rheumatoid arthritis and systemic lupus erythematosus. The disease burden posed by fibrosis‐related disorders is enormous, being a significant cause of morbidity and mortality in chronic inflammatory diseases, accounting for about 50% of deaths globally [3, 4]. Besides, effective treatment methods are not readily available. Many factors contribute to the development of chronic fibrotic disease. Irrespective of the contributing factor, a common feature of fibrotic‐associated diseases is the hyperactivation of ECM‐producing cells (myofibroblasts), the main driver of fibrotic tissue remodelling in wound healing and tissue repair [5, 6].

Poikiloderma with tendon contracture, myopathy and pulmonary fibrosis (referred to as POIKTMP, OMIM #615704) is a rare autosomal dominant hereditary multi‐organ fibrosing disorder with about 40 cases reported worldwide since it was first described by Khumalo et al. in 2006, in a South African family of European descent [7, 8]. This disease, which first presents as scleroderma (skin fibrosis or scarring) in patients among other organs, including skeletal muscles, eventually leads to lung complications (pulmonary fibrosis) that lead to death. Subsequently, mutations in the *family with sequence similarity member 111 B* (*FAM111B*) gene were described as the genetic basis for the disease [9]. About 20 pathogenic and non‐pathogenic variants of this gene have been reported, with roughly ten related to POIKTMP (including the *FAM111B Y621D* mutation identified in the South African family first diagnosed with POIKTMP) [10, 11].

Although published studies have aided our understanding of the human *FAM111B* gene and its contribution to POIKTMP and cancers, only a handful have investigated the molecular changes associated with *FAM111B* mutations relating to inflammation and fibrosis, given that both are histological features of POIKTMP. Therefore, this study examined the pathological changes in genetic and gene expression profiling of the *FAM111B* gene and fibrosis‐related genes in post‐mortem tissues of POIKTMP.

## 2 Materials and Methods

### 2.1 Ethics and Study Samples

The study obtained Ethical Clearance from the University of Cape Town, with the reference number HREC 057/2021. Formalin‐fixed and paraffin‐embedded (FFPE) post‐mortem tissue sections (skin, lung and skeletal muscle) from a member of the South African family affected by POIKTMP and three age and gender‐matched healthy non‐familial controls were studied. The causes of death for the control individuals were unrelated to fibrotic or connective tissue disorders and were primarily due to unnatural causes (e.g., trauma or accidental deaths).

### 2.2 Sample Preparation for Nucleic Acids and Protein Extraction

FFPE tissue sections (3 × 10 μm) were deparaffinised with xylene using standard dewaxing procedures. Next, the samples were rehydrated using a graded ethanol system (100%, 95% and 70%). The rehydrated tissues were incubated at 37°C for 10 min to remove residual ethanol and were subsequently used to isolate nucleic acids and proteins.

### 2.3 DNA Extraction

Genomic DNA was isolated from the dewaxed and rehydrated tissues using the QIAamp DNA FFPE Tissue kit (Cat. No. 56404, Qiagen) based on the kit's manufacturer's protocol. The eluted DNA was quantified using a nucleic acid concentration measuring instrument (Biodrop, Biochrom, WhiteSci).

### 2.4 RNA Extraction and Gene Expression Studies

Total RNA from the FFPE sections was dewaxed and deparaffinised. The extraction was performed using the RNeasy FFPE kit (Cat. No. 73504, Qiagen, Germany), following the manufacturer's guidelines for RNA recovery. The RNA quantity and quality were verified using a nucleic acid analysis instrument (i.e., Biodrop).

For transcript expression analysis, 10 ng of purified total RNA was reverse transcribed (cDNA synthesis) using the LunaScript RT SuperMix kit (Cat. No. E3010S, New England BioLabs). The total RNA samples for the healthy control patients were pooled because they were from the same population to reduce costs. The cDNA synthesis reaction was incubated using the T100 Thermal Cycler (Cat. No. 1861096, BioRad) using the cycling conditions prescribed by the kit's manufacturer. The expression of all target genes in the synthesised cDNA was determined by quantitative polymerase chain reaction (qPCR) using the Luna Universal qPCR Master Mix (Cat. No. M3003, New England BioLabs). Specific primers for the quantified genes, including *FAM111B* and control housekeeping genes, *GAPDH* and *ACTIN*, are listed in Table S1, and the qPCR reaction was performed using the QuantStudio 6 Flex (Cat. No. 4485699, Applied Biosystems). Each sample (POIKTMP patient or pooled control) was run in triplicate wells, and each experiment was repeated three times independently using the same RNA samples. Thus, all replicates in this study represent independent technical repeats, not additional biological samples. The relative gene expression was calculated using the 2^−ΔΔCT^ method [12, 13]. The *FAM111B* expression was normalised to *GAPDH* and *β‐ACTIN* housekeeping genes.

### 2.5 Validating *FAM111B Y621D* Genotype in POIKTMP Tissues

The *FAM111B Y621D* patient mutation was genotyped by performing Sanger Sequencing using the extracted DNA. The extracted DNA was first amplified by PCR using primers targeting the *FAM111B* (Table S1) and the Platinum PCR Universal Master Mix (Cat. No. A44647100, ThermoScientific). The amplification was verified by running a sample of the amplicon on a 1% agarose gel and electrophoresis, followed by visualisation on an Azure c400 Geldoc imaging system (Azure Biosystems, United States). The remainder of the amplicon was then purified using the GeneJet PCR Purification kit (Cat. No. K0702, Thermo Scientific). The Sanger sequencing of the purified amplicon was performed as a service at the University of Stellenbosch's Central Analytical Facilities (CAF), and the sequencing results aligned with the canonical *FAM111B* gene sequence (NM_198947.4) using the alignment tool of the SNAPgene software (GSL Biotech LLC, USA) to identify the *Y621D* mutation.

### 2.6 FAM111B Gene Expression, Human Fibrosis Gene Profiling and Target Validation

These experiments were performed using the catalogued Format A reagents of the RT^2^ Profiler PCR for human fibrosis Array kit (Cat. No. PAHS‐120Z, Qiagen) compatible with the QuantStudio flex 6 (Applied Biosystem, Life Technologies) real‐time cycler. 25 ng of purified RNA samples from the pooled control groups and the same amount of RNA from the patient's sample were quantified, and quality control was verified using the Agilent 2100 Bioanalyser (Cat. No. G2939BA, Agilent) with the RNA 6000 Nano Lab Chip. The cDNA was synthesised using the RT^2^ First Strand Synthesis Kit (Cat. No. 330401, Qiagen), and the targets were quantified using the RT^2^ SYBR Green qPCR Mastermix (Cat. No. 330529, Qiagen). RT^2^ Profiler PCR Array for Human Fibrosis Genes (Cat. No. PAHS‐120Z, Qiagen, Germany) contains 84 pathway or fibrosis pathway‐focused genes and 5 housekeeping genes (*ACTB*, *B2M*, *GAPDH*, *HPRT1* and *RPLP0*) in addition to genomic DNA control, 3 reverse transcription controls, and 3 positive PCR controls. The cycle threshold (Ct) values in the RT‐PCR were used to measure the target gene amplification and expression. Each sample array experiment was repeated three times independently using the same RNA samples. Thus, all replicates in this study represent independent technical repeats. The cycle threshold (Ct) values were then exported into an Excel file and uploaded to the GeneGlobe data analysis web portal at https://geneglobe.qiagen.com/. Samples were assigned to controls and test groups. The Ct values were normalised based on the reference genes, and the fold change of the expressed genes was calculated using the delta–delta Ct method of the GeneGlobe data analysis web portal. The analysed data (fold change), including the relevant visualisations for the differentially expressed genes, were then exported as an Excel or PDF document for further analysis.

From the data analysis reports, some of the top differentially expressed genes were selected and validated with qPCR using the targeted primer listed in Table S1. The Ct values were normalised against those of the housekeeping genes: *GAPDH* and *β‐ACTIN*. The stability of the housekeeping genes was established by comparing the Ct values in both the control and patient samples without any significant variation (i.e., ΔCt < 0.5 and *p* ≥ 0.05), and the fold change of the expressed genes was calculated using the delta–delta Ct method.

### 2.7 Disease and Pathway Enrichment Analysis

#### 2.7.1 Network Analysis and Enrichment Analysis

Disease and pathway enrichment analysis was conducted using the genes of interest to identify potential associations with various diseases and biological pathways. Although lung and skin tissues exhibited distinct gene expression profiles, their datasets were combined for enrichment analysis to capture overlapping fibrotic pathways and shared molecular networks. This approach reflects the systemic, multi‐organ nature of POIKTMP, while the tissue‐specific differences are presented separately in Figures 2, 3, 4, 5.

The enrichment analysis was primarily performed using Metascape [14], which integrates multiple curated databases, including Gene Ontology (biological process, molecular function and cellular component), KEGG Pathway, Reactome, BioCarta/MSigDB canonical pathways, DisGeNET (disease associations), TRRUST (transcription factor–target interactions), and CORUM (protein complexes). Metascape applies a hypergeometric test with multiple‐testing correction to determine significantly enriched biological themes. The enrichment factor was calculated as the ratio of observed gene counts to those expected by chance, and terms meeting the criteria of *p* < 0.01, minimum count ≥ 3, and enrichment factor > 1.5 were grouped into clusters based on membership similarity. The network relationships among the differentially expressed genes were further evaluated using GeneMANIA as described previously [15, 16, 17]. GeneMANIA integrates co‐expression, protein–protein interactions, and functional similarity data to predict gene–gene associations. Additional enrichment analyses were conducted using Enrichr, which applies Fisher's exact test and the hypergeometric test to generate combined scores across diverse libraries [18]. The enrichment results were then processed and visualised in MATLAB (2023, Version 9.14, R2023b; The MathWorks Inc., Natick, Massachusetts), and the top diseases and pathways were identified based on their combined scores.

#### 2.7.2 Interactome Network Construction

Multiple data sources were integrated to create a comprehensive protein–protein interaction network, including the UCSC Super Pathway, ChEA database, KEA database, and BioGRID. The UCSC Super Pathway [19] was obtained from a text file, while the ChEA [20] and KEA interactions [21] were extracted from their respective Excel files. The BioGRID interactions were retrieved using the BioGRID Application Programming Interface. The data were filtered to include only human proteins and evidence from Affinity Capture‐MS and Two‐hybrid experiments to ensure only high‐confidence interactions, eliminating potential false positives.

### 2.8 Statistical Analysis

All statistical analyses were performed with GraphPad Prism Version 9.0.0 (GraphPad, United States). Statistical significance was defined as a *p*‐value ≤ 0.05 when comparing the mean and ± SEM between the control and patient sample groups as determined by the *t*‐test.

## 3 Results

### 3.1 *FAM111B* Gene Is Differentially Expressed in Patient Versus Control Tissues

**FIGURE 1:**
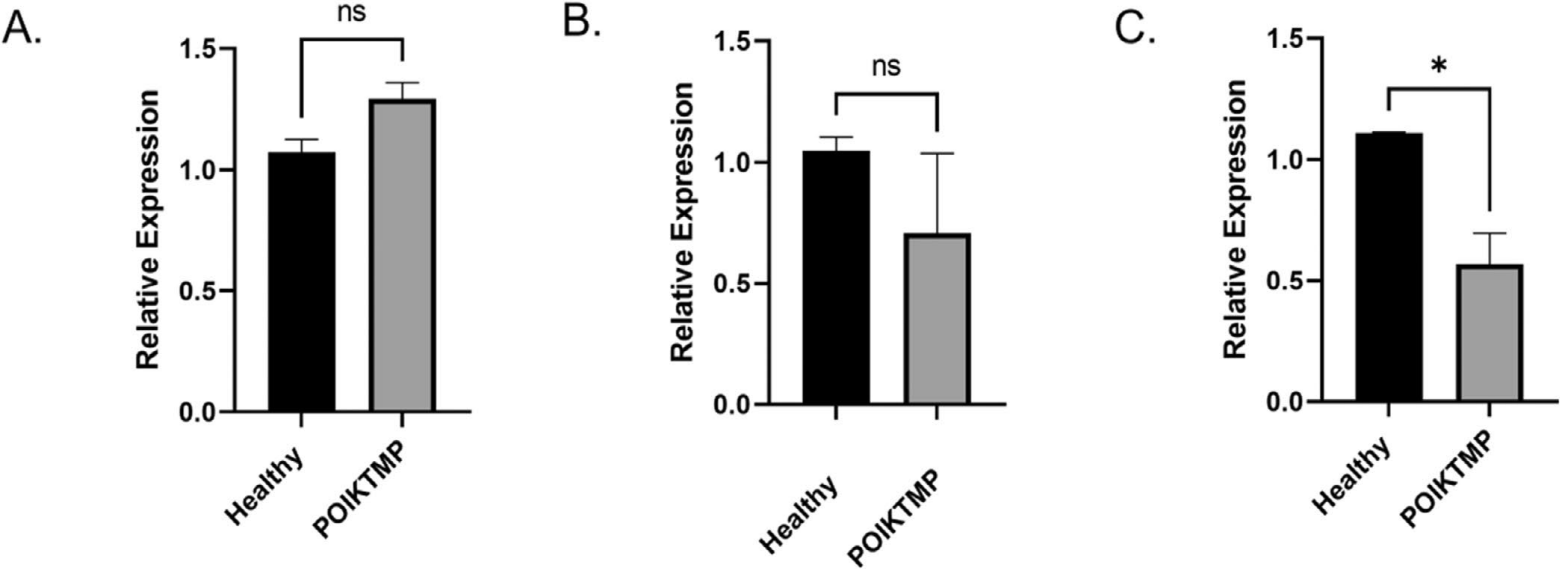
A comparative gene expression of the human *FAM111B* gene in healthy controls and POIKTMP tissues of the (A) skeletal, (B) lungs and (C) skin. The relative gene expression was quantified using qPCR, and *FAM111B* expression was normalised to *GAPDH* and *β‐Actin* housekeeping genes. Statistical analysis was performed using a *t*‐test, and statistical significance was determined by a *p*‐value (*) ≤ 0.05; ns (non‐significant). All qPCR reactions were run in triplicate wells, and each experiment was repeated three times independently using the same RNA samples (technical replicates).

Following the validation of the presence and absence of the *FAM111B Y621D* mutation in the extracted DNA of POIKTMP patient and healthy control tissues, respectively (Figure S1) by Sanger Sequencing, the gene expression of *FAM111B* was quantified by qPCR using mRNA isolated from the same tissue samples. The qPCR gene expression showed that *FAM111B* was expressed in the lungs, skin and skeletal muscle of the POIKTMP patient with the *FAM111B Y621D* mutation. Although there was no difference in the expression of *FAM111B* in the patient compared to control tissue samples of the skeletal muscle and lungs (Figure 1A,B), the expression was about two‐fold lower in the patient's skin tissues than in the controls (Figure 1C).

### 3.2 Specific Fibrosis‐Related Genes Are Differentially Expressed in Patient Versus Control Tissues

**FIGURE 2:**
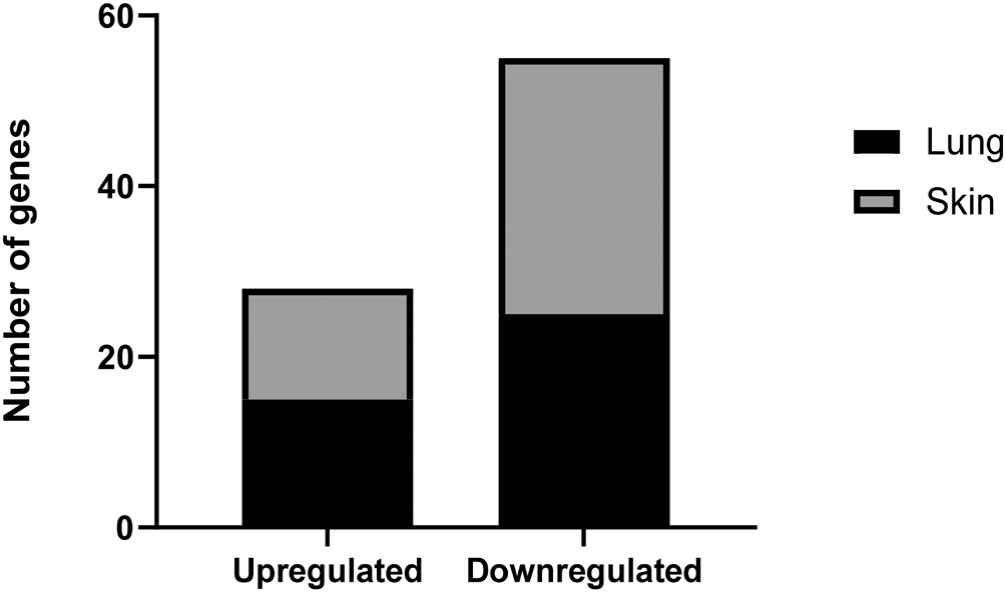
Differentially expressed fibrosis genes in the skin and lung tissues of a POIKTMP patient versus pooled healthy control tissues using the RT^2^ Profiler PCR Array of Human Fibrosis panel of genes. The estimated number of genes from the panel of 84 pathway‐focused fibrotic genes, with changes in relative expression in the lung and skin of POIKTMP patients compared to the healthy control. In total, 83 genes were differentially expressed: 28 upregulated (15 in lung, 13 in skin) and 55 downregulated (25 in lung, 30 in skin). Each array experiment was repeated thrice independently using the same RNA samples (technical replicates, *n* = 3).

Differential gene expression of human fibrosis markers was only conducted in the POIKTMP patient's skin and lung tissues. Of the 84 fibrosis‐associated genes represented on the RT^2^ Profiler PCR Array, 83 were differentially expressed across lung and skin tissues. A total of 28 genes were upregulated (15 in lung and 13 in skin), while 55 genes were downregulated (25 in lung and 30 in skin) compared with pooled controls (Figure 2).

A comparison of gene expression in POIKTMP lung and skin tissues to normal tissues revealed several upregulated and downregulated genes. The upregulated genes included *CTGF, PDGFRA, ITGB1, THBS2, COL3A1, MMP13, MMP3 and TGFB3*. The downregulated genes were *ACTA2, PLAT, THBS1, LOX, MMP8, MMP13 and CXCR4*.

**FIGURE 3:**
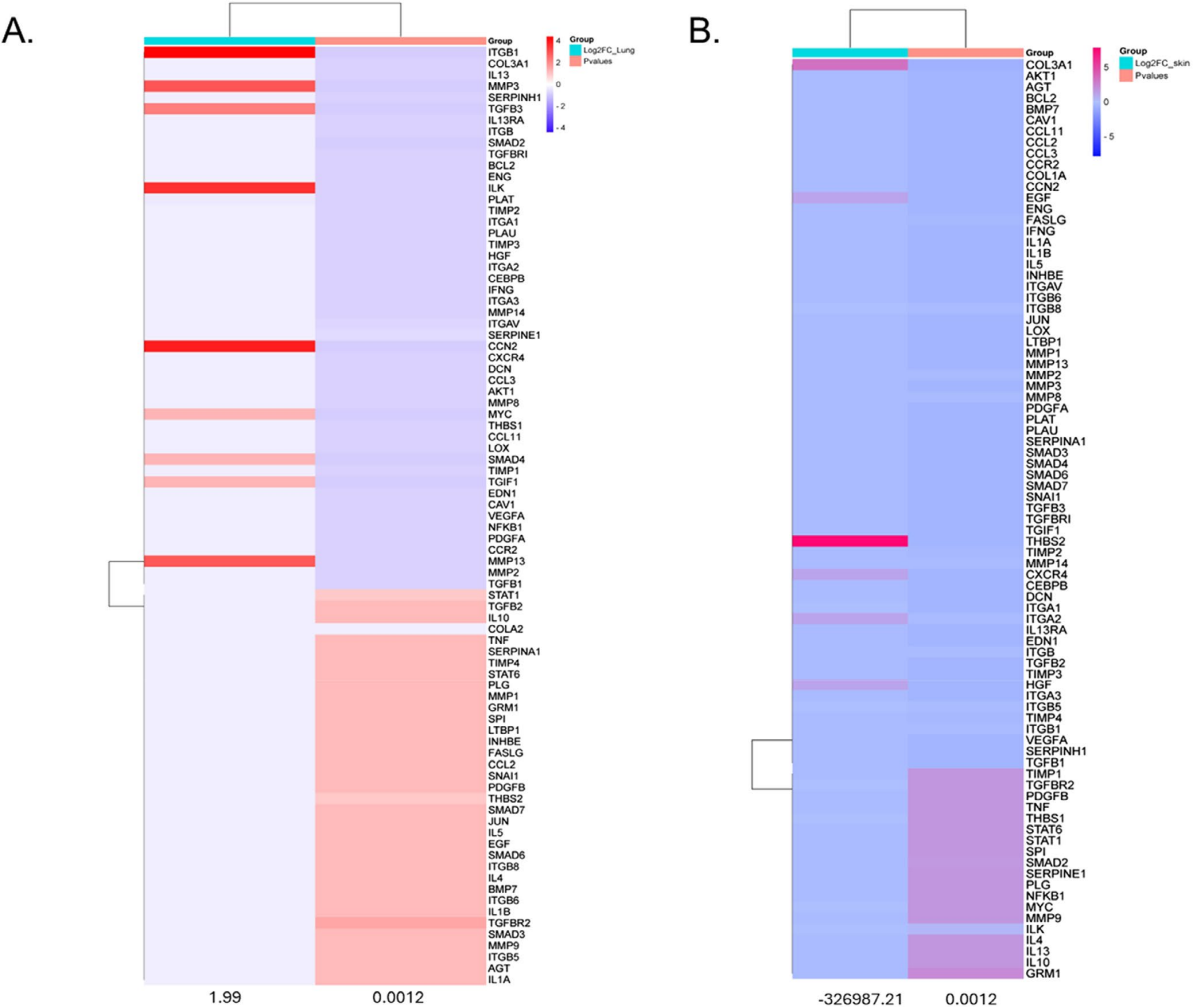
Heat map plots showing gene expression profiles for 84 fibrosis‐associated genes in (A) lung and (B) skin tissues from a POIKTMP patient versus pooled controls. The colour scale represents log_2_ fold change (Log_2_FC): Red = upregulated, blue = downregulated, white = minimal change. Statistical significance (*p* ≤ 0.05) is defined by intensity of shading: Darker tones correspond to lower (more significant) adjusted *p*‐values. Not all genes shown are statistically significant. The x‐axis represents numeric ticks used for the plotting scale and does not denote categorical sample labels.

The heat map plots provide an overview of expression changes across all 84 fibrosis‐associated genes in lung and skin tissues (Figure 3). Values are presented as log_2_‐transformed fold changes (Log_2_FC), with red indicating upregulation, blue indicating downregulation, and white/pale indicating minimal change. The statistical significance (*p* ≤ 0.05) of these changes is shown by colour intensity: darker shades correspond to lower (more significant) adjusted *p*‐values. It's important to note that the maps highlight the overall distribution of tissue expression changes and don't imply that all genes are statistically significant. For example, in the POIKTMP lung tissue, fibrotic markers such as *integrin beta‐1 (ITGB1), matrix metalloproteinase‐1 (MMP1), matrix metalloproteinase‐3‐1 (MMP3), matrix metalloproteinase 3 & 13 (MMP3 & MMP13), transforming growth factor beta‐3 (TGFβ‐3), connective tissue growth factor (CCN2), and platelet‐derived growth factor subunit A (PDGFA)* were strongly upregulated (Figure 3A). In the skin, *Collagen alpha‐type 111 (COL3A1)* and *Thrombospondin 2 (THBS‐2)* were strongly upregulated (Figure 3B).

### 3.3 Validation of the Top‐Regulated Fibrotic Genes

**FIGURE 4:**
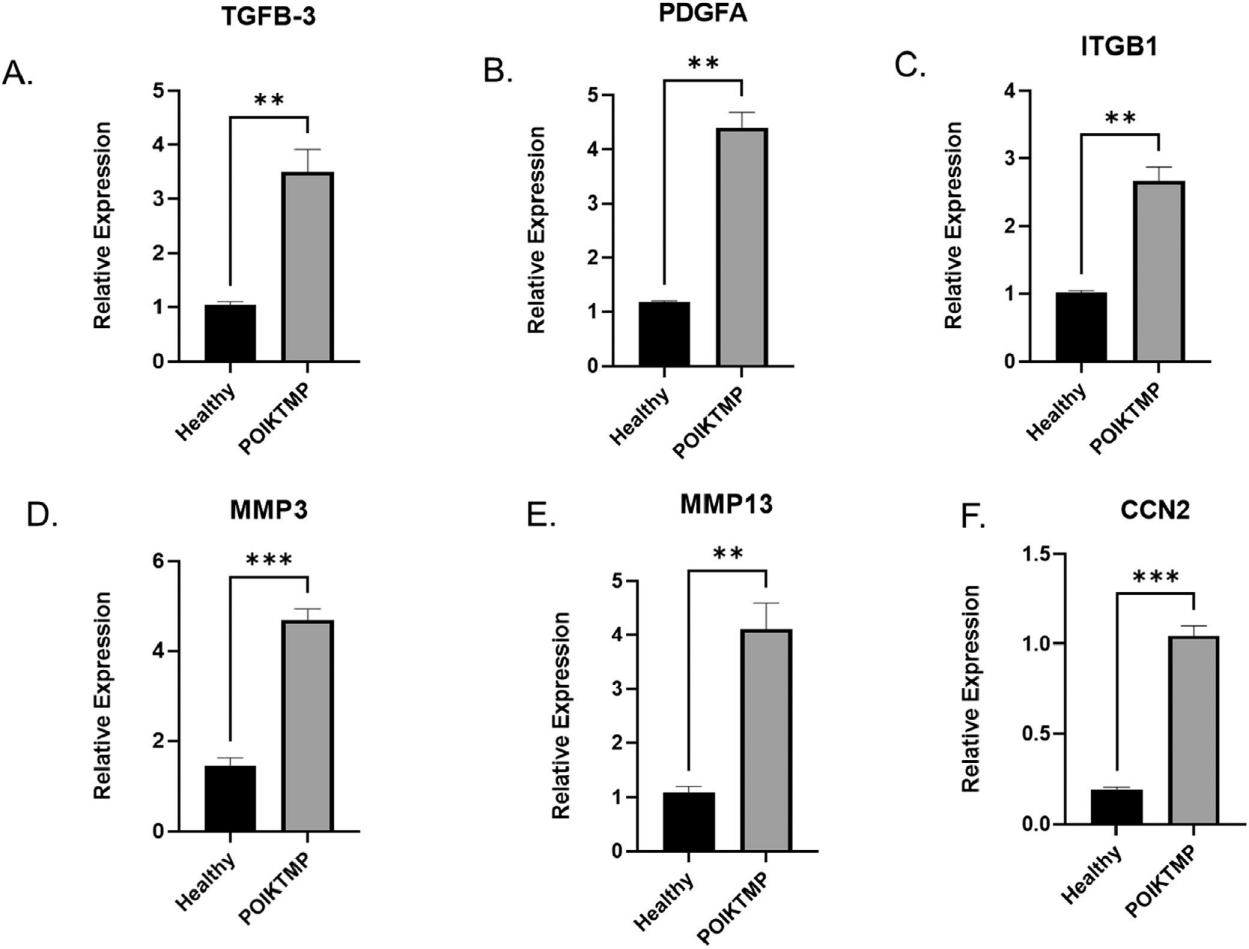
Validation of the top‐upregulated fibrosis genes in the lung tissue by qPCR (A) *TGFβ‐3*; (B) *PDGFA*; (C) *ITGB1*; (D) *MMP3*; (E) *MMP13*; (F) *CCN2*. Relative gene expression was higher in the upregulated genes of interest in the patient's tissue for all markers. Quantification was relative to the familial healthy control mRNA expression levels normalised to *GAPDH* and *β‐Actin*. All experiments were performed in triplicate and repeated three times with similar results. Statistical analysis was performed using *t*‐test (*p ≤ 0.05; **p ≤ 0.001; ***p ≤ 0.0001).

**FIGURE 5:**
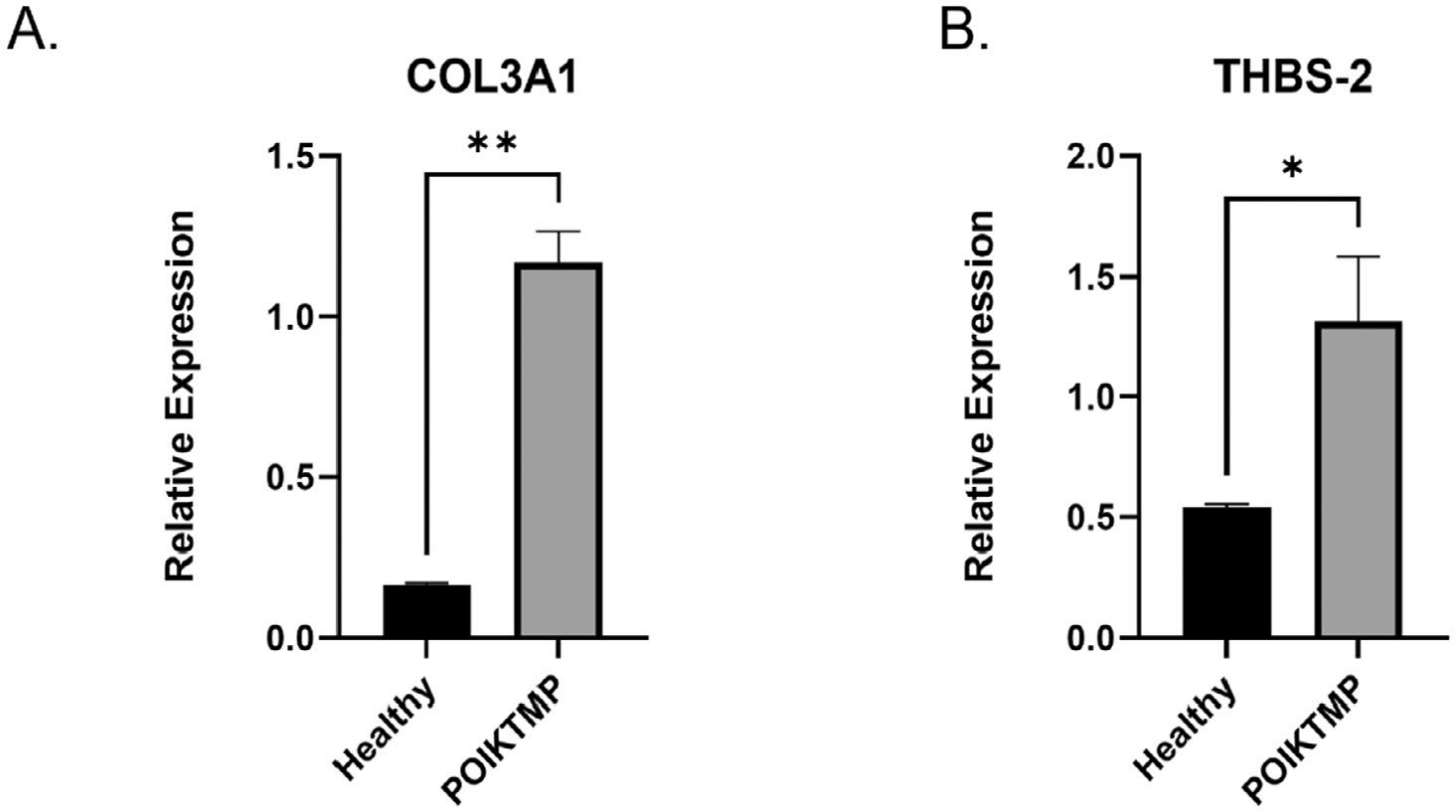
Validation of the top‐upregulated fibrosis gene in the skin tissue. (A) *COL3A1*; (B) *THBS‐2*. Relative gene expression was higher in the upregulated genes of interest in the patient's tissue for the markers quantified. Quantification was relative to the familial healthy control mRNA expression levels normalised to *GAPDH* and *β‐Actin*. All experiments were performed in triplicate wells and repeated three times using the same RNA samples (technical replicates). Statistical analysis was performed using a *t*‐test (*p ≤ 0.05; **p ≤ 0.005).

The expressions of these top‐regulated human fibrosis‐related gene markers identified in POIKTMP lung were validated using qPCR. As indicated by the RT^2^ Profiler PCR array analysis report for Human fibrosis exported from GeneGlobe, the genes *TGFβ‐3*, *PDGFA, MMP13, MMP3, CCN2* and *ITGB2* were highly expressed in the lung tissue of the POIKTMP patient compared to the healthy familial control (Figure 4). Likewise, the *COL3A1* and *THBS2* genes were significantly higher in the skin tissue of the POIKTMP patient than in the healthy control (Figure 5). This data indicates that the POIKTMP and possibly the *FAM111B* Y61D mutation affect the expression of other fibrotic genes, suggesting additional functions and pathways associated with *FAM111B*.

### 3.4 Disease, Gene and Pathway Enrichment Analysis

#### 3.4.1 Network Analysis and Enrichment Analysis

**FIGURE 6:**
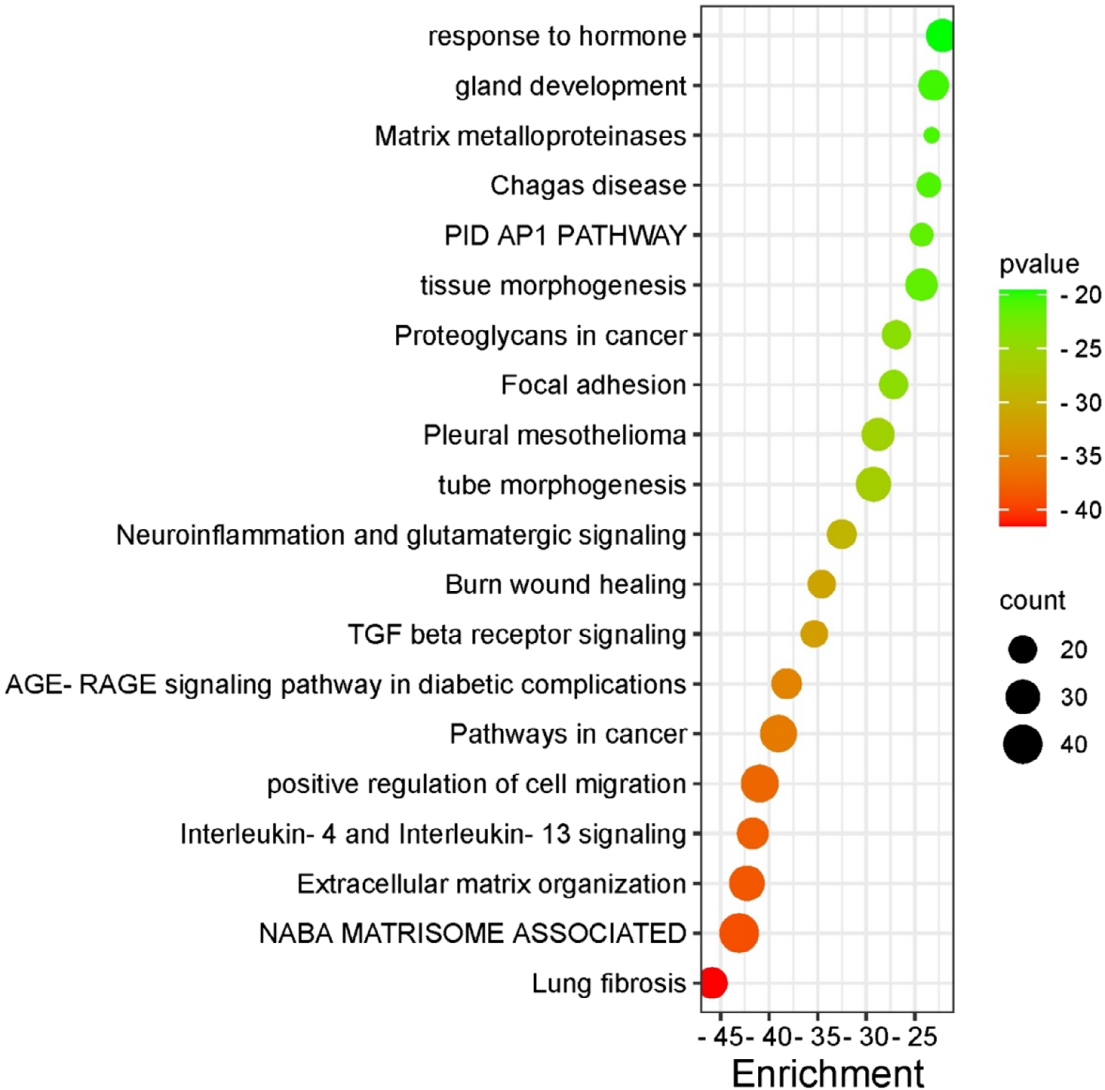
Top 20 clusters with representative enriched terms identified using Metascape. Enrichment analysis was performed with a hypergeometric test and multiple testing correction across curated databases (GO, KEGG, Reactome, MSigDB, DisGeNET, TRRUST, CORUM). Terms with *p*‐value < 0.01, minimum count of 3, and enrichment factor > 1.5 are grouped into clusters based on membership similarities. Enrichment scores are shown as −log_10_ (*p*‐value), where larger values indicate stronger significance.

The gene set enrichment analyses with Metascape showed the Top 20 clusters with their representative enriched terms. Metascape combines functional enrichment, interactome analysis, gene annotation and membership search to leverage over 40 independent knowledge bases within one integrated portal. In the study, 40 genes, including the *FAM111B* gene, are strongly associated with cancer pathways, cellular migration regulation, Nuclear and Apical Basal Axis (NABA) microsome, extracellular matrix organisation and lung fibrosis (Figure 6). Additionally, the identification of these interactomes suggests potential diagnostic and druggable targets with *FAM111B*. Many gene annotation pathways and protein interaction databases are primarily compiled for human genes/proteins. Gene enrichment analysis using Metascape provides access to the most extensive curated databases, enabling answers to common queries such as which pathways or biochemical complexes are enriched and the functional roles of the identified protein complexes. It also informs whether candidate proteins are secreted, contain a transmembrane domain, or are otherwise druggable, or if there are chemical probes available for rapid candidate validation. Critically, when multiple gene lists are analysed, either from common or orthogonal platforms, identifying consistent underlying pathways or networks can help decipher authentic signals above the experimental noise.

**FIGURE 7:**
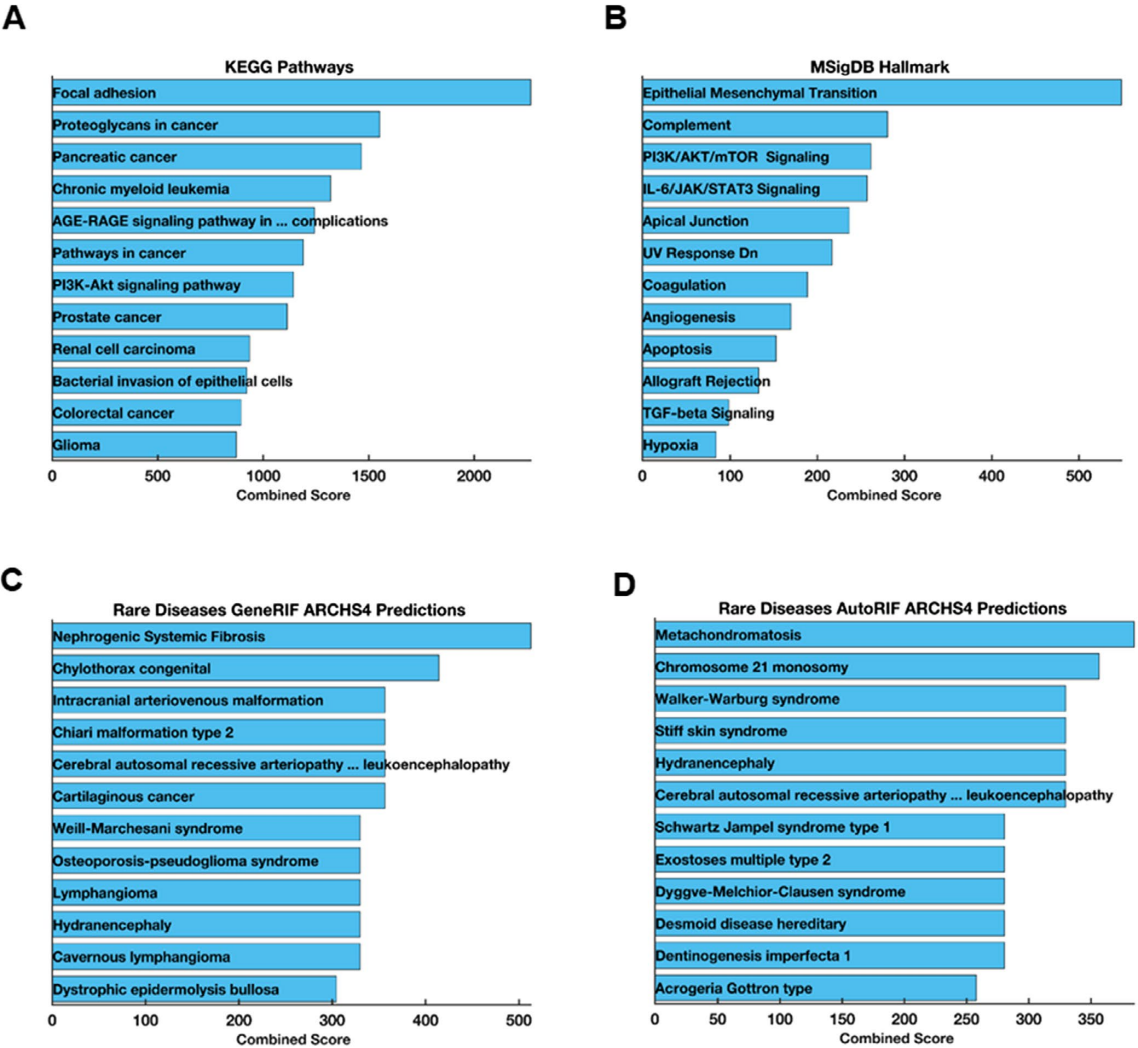
Disease and Pathway Association of *FAM111B*. (A) KEGG pathway enrichment analysis for the genes strongly interacting with the selected gene list. (B) The Human Molecular Signatures Database (MSigDB) of the selected gene set list. (C) Rare Diseases AutoRIF ARCHS4 prediction gene‐set library. (D) Rare Diseases GeneRIF ARCHS4 prediction of selected gene‐set library lists consisting of up and downregulated genes associated with FAM111B.

Furthermore, the diseases, pathway enrichment analysis, and protein–protein interaction results corroborate the biological process, cellular process and molecular functions of the upregulated genes in this study (Figure 7, Figure S2). Furthermore, we found significant associations between the genes of interest and pathways such as pathways in cancer (*p*‐value: 4.48e−58, combined score: 1190.1), focal adhesion (*p*‐value: 2.36e−55, combined score: 2268.1), PI3K‐Akt signalling pathway (*p*‐value: 5.94e−49, combined score: 1142.9), proteoglycans in cancer (*p*‐value: 3.60e−46, combined score: 1552.1), and human cytomegalovirus infection (*p*‐value: 1.22e−33, combined score: 790.47). Top diseases identified in our study include cancers (*p*‐value: 6.18e−25, combined score: 381.92), arthritis (*p*‐value: 2.90e−19, combined score: 327.29), lung disease (*p*‐value: 3.42e−17, combined score: 360.87), cerebrovascular disease (*p*‐value: 2.67e−13, combined score: 152.60), and breast cancer (*p*‐value: 2.43e−12, combined score: 97.14) (Figure S2).

#### 3.4.2 Interactive Networks Associated With *FAM111B*

The constructed interactome network integrated data from multiple sources, revealing potential protein–protein interactions among the upregulated and downregulated genes in POIKTMP skin and their interactions with *FAM111B*. The *FAM111B* network, consisting of 24 edges and 25 nodes, suggests a central role for *FAM111B* in the molecular processes associated with POIKTMP skin. The upregulated genes network has 719 edges and 503 nodes, and the downregulated genes network has 434 edges and 295 nodes (Figure S3A,B). The visual representation of the FAM111B network showed distinct interactions between upregulated and downregulated genes and their connections to *FAM111B*. The network properties indicated a highly connected network, with some proteins acting as hubs for multiple interactions (Figure S4). The results showed that the genes have 63.05% co‐expression, 12.38% physical interactions, 8.10% shared protein domains, 4.70% co‐localization, and only 7.80% shared similar biological pathways. Some of the top biological functions identified with strong *p*‐values (7.29 × 10^−9^–7.72 × 10^−11^) are negative regulation of peptidase activity, regulation of oxidoreductase activity, negative regulation of proteolysis, regulation of blood vessel diameter, regulation of tube size, vasoconstriction and extracellular matrix organisation. This connectivity suggests that FAM111B and its interactors may play crucial roles in the pathogenesis of POIKTMP skin and potentially other related diseases.

## 4 Discussion

The study used tissue samples from post‐mortem FFPE tissue sections emanating from a member of the South African family first diagnosed with POIKTMP. The skin and lungs (and, to a lesser extent, skeletal muscle) are the tissues most severely affected by the POIKTMP disease [9, 22]. Furthermore, this family was part of the cohort whose DNA samples were sequenced by Whole‐exome sequencing, which identified mutations, including the *Y621D* mutation, in the *FAM111B* gene as the causative gene for the POIKTMP syndrome [9].

Gene expression profiling is effective in discovery analysis experiments like RT‐PCR or qPCR, microarray and RNA sequencing that allow the description of genome‐wide expression changes in disease and healthy states. One such method, the RT^2^ PCR Array Profiler, combines RT‐PCR and microarray techniques to help compare gene expression levels among conditions [23, 24]. *FAM111B* mRNA expression has been reported in many tissues, such as skin, skeletal muscle and trachea [25]; recent experimental studies have detected mRNA expression in lung cancer cell lines and skin fibroblasts [9, 11]. The present study sought to assess *FAM111B* expression in tissue types affected by POIKTMP and critical genes associated with fibrosis.

RT‐qPCR was used to measure *FAM111B* relative gene expression in the skeletal lungs and skin tissues. Although *FAM111B* mRNA expression was not differentially expressed in the lungs and skeletal muscle of POIKTMP patients' tissues compared to the controls, expression was seen to be downregulated in the patient's skin tissues. Similar results were seen in POIKTMP patient‐derived skin fibroblasts, which showed a decrease in *FAM111B* expression [11]. FAM111B mutations, beyond just differential expression, cause excessive protease activity, disrupting cellular processes and triggering apoptosis. This cytotoxicity arises from the dysregulation of the FAM111B gene product and its inherent serine protease‐like activity. This information implies that the presence of the mutation may impact gene expression levels and function. Also, the presence of the *FAM111B* gene mutations within its protease domain has been proposed to contribute to disease severity, expression and phenotypic variations [10]. While these gene‐level findings are informative, protein‐level validation would have significantly strengthened the study's conclusion. However, protein validation was not feasible given the nature of the FFPE tissues used in this study (post‐mortem and archival), as protein extraction of sufficient quality and quantity for western blotting was not achievable. Nonetheless, future studies will employ fresh tissue samples or patient‐derived cell lines, incorporating protein‐level validation of FAM111B and its downstream targets.

This study used the RT^2^ Profiler PCR Array Human Fibrosis system, which contains a panel of 84 fibrosis pathway‐associated genes, to profile the gene expression of fibrotic markers in POIKTMP‐affected tissues. Since POIKTMP is a multisystem fibrosing disease implicated in fibrosis, the study used skin and lung tissues, the main organs affected by POIKTMP [9]. Eight of the 84 pathway‐focused fibrosis genes assessed were upregulated in the POIKTMP patient‐associated tissues (6 in lungs and 2 in skin). In the POIKTMP lung tissue, pro‐fibrotic markers *platelet‐derived growth factor subunit A (PDGFA)*, *matrix metalloproteinase 3 and 13 (MMP3 & MMP13)*, *integrin beta‐1 (ITGB1), transforming growth factor beta‐3 (TGFβ‐3)*, and *connective tissue growth factor (CCN2)* were upregulated. *Collagen alpha‐type III (COL3A1) and thrombospondin 2 (THBS‐2)* were highly upregulated in the skin. These pro‐fibrotic markers belong to categories of growth factors involved in signal transduction, inflammatory cytokines and chemokines, epithelial to mesenchymal transition (EMT) genes, ECM and cell adhesion molecules of fibrosis. A key pro‐fibrotic mediator upregulated in this experiment is *TGFβ‐3*, a critical marker that promotes the formation of ECM fibroblasts, myofibroblast differentiation and resistance to apoptosis. Overexpression of *TGFβ‐3* in vivo has been reported to cause severe fibrosis in experimental animal fibrosis models and human studies of end‐stage tissue fibrosis despite the involvement of several complex signalling pathways. Also, previous *FAM111B‐*related studies have implicated *TGF‐β1* to be associated with POIKTMP [7, 9], which is also analysed in the current protein expression study.

ECM remodelling enzymes and cell adhesion molecules, *MMP3* and *MMP13*, were identified as being highly regulated in the patient lung tissue in this study. Several MMP isoforms have been identified as degraders of various substrates, including ECM components such as collagens, elastin, proteoglycans and lamins [26, 27]. The multiple biological roles of MMPs suggest that they play a role in a wide range of pathological processes, including rheumatoid arthritis, tumour progression, kidney fibrosis, liver fibrosis, heart failure and lung fibrosis [26, 28]. *MMP3* and *MMP13* have also been shown to contribute to the pathogenesis of pulmonary fibrosis in humans and animal models [28]. Interestingly, this study has observed all these mediators, which could support their role in pulmonary fibrotic diseases.

The *CCN2* and *PDGFA* expression was also upregulated in the POIKTMP patient lung tissue, and both mediators have been demonstrated to stimulate fibroblast proliferation in vitro [29]. Additionally, the overexpression of *CCN2* has been reported in an in vivo bleomycin‐induced skin fibrosis model [29]. *PDGFA* is upregulated in animals with fibrosis. In association with *TGF‐β*, crosstalk between the two mediators has been proposed to cause fibroblast proliferation [30, 31]. Interestingly, this association between the two growth factors enhances apoptosis in epithelial cells and has been reported to resist apoptosis in mesenchymal cells [32]. *COL3A1* and *THBS‐2* showed high expression in the POIKTMP patient's skin tissue; both are ECM molecules, specifically non‐structural matricellular proteins. Systemic sclerosis (SSc) fibrosis is profoundly influenced by dysregulated processes promoting fibrosis. SSc fibrosis is a rare chronic heterogeneous disease characterised by inflammation and vasculopathy and has also been associated with *THBS‐2* [30]. As with *FAM111B* in POIKTMP, SSc leads to multisystem tissue fibrosis during the second decade of affected persons' lives as disease severity progresses. Healthy and fibrotic lungs contain a balanced amount of collagen, a component of lung connective tissue. Idiopathic pulmonary fibrosis, however, is characterised by excessive *COL3A1* production facilitated by the presence of myofibroblasts [33]. By highlighting these essential genes, this data supports the association of *FAM111B* with *TGF‐β3*, along with other mediators of fibrosis. Although most studies examining *FAM111B* gene mutations have found its expression downregulated, the current research focuses on the highly expressed genes of interest among the 84‐panel‐focused fibrotic genes. This study suggests that the pro‐fibrotic markers affect the ECM turnover during fibrosis, cellular proliferation and inflammatory responses. *FAM111B* is reported to activate myofibroblast activation directly and indirectly, supporting its involvement in fibrosis [34]. *COL3A1* and *THBS‐2* have also been associated with Ehlers‐Danlos Syndrome (EDS), a group of heritable connective tissue disorders characterised by joint hypermobility, fragile skin, and, in some cases, a high risk of vascular complications due to weakened blood vessel walls [35, 36, 37, 38, 39]. Intriguingly, similar pathological skin phenotypes are seen in POIKTMP patients. Mutations in *COL3A1*, which encodes type III collagen, are a hallmark of Vascular EDS (vEDS), the most severe form, known for spontaneous arterial dissections and ruptures [39]. *THBS‐2*, encoding thrombospondin‐2, is crucial in maintaining extracellular matrix integrity. A recent study identified a specific *THBS‐2* mutation associated with a novel form of EDS with prominent vascular features, including joint hypermobility, prolonged bleeding and risk of aortic complications [36]. Further investigation is needed to explore the functional consequences of *COL3A1* and *THBS‐2* upregulation in POIKTMP and elucidate potential links to the observed phenotype, particularly regarding myofibroblast activation and ECM remodelling.

Importantly, the transcriptomic alterations identified in this study reflect the patient's clinical presentation. The patient developed progressive pulmonary fibrosis, consistent with the strong upregulation of *TGFβ‐3, PDGFA, ITGB1, MMP3, MMP13 and CCN2* in lung tissue, all of which are established drivers of extracellular matrix deposition, fibroblast proliferation and tissue remodelling [7, 28, 29, 30, 31, 32]. Similarly, the increased expression of *COL3A1* and *THBS2* in skin tissue correlates with the patient's cutaneous fibrosis and scleroderma‐like phenotype [7, 30, 33, 36]. These molecular changes not only reinforce the pathological features observed in POIKTMP but also highlight how FAM111B dysregulation may influence specific fibrotic pathways that manifest clinically in skin and lung disease.

Additionally, gene‐set enrichment analysis (GSEA) was used in this study to elucidate the association between *FAM111B* and the list of upregulated genes obtained from the RT^2^ Profiler PCR Array Human fibrosis analysis. The study further implicates *FAM111B* and other genes of interest as significant causes of POIKTMP in the KEGG biological pathway (Figure 7A); precisely, the focal adhesion pathway was highly predicted. While it remains to be proven that the focal adhesion pathway is disrupted in POIKTMP, this phenomenon has been shown in EDS and Hypermobility Spectrum Disorders (HSD), which have overlapping clinical features with POIKTMP [38, 40], and thus warrants further investigation. The enrichment of the proteoglycans pathway in cancer, including pancreatic cancer, supports previous knowledge of FAM111B's association with various cancers, particularly pancreatic cancer. However, it can be argued that *FAM111B* contributes to prostate cancer susceptibility through multiple single‐nucleotide polymorphisms (SNPs) located on 11q12, which is in the same chromosomal position as *FAM111B* and other *FAM111* member genes [41]. Moreover, evidence in lung adenocarcinoma patients of *FAM111B* suggests its strong correlation with tumour progression, metastasis and poor survival rates [42, 43]. Additionally, *FAM111B* has been linked to different types of cancers, including pancreatic, liver and breast cancers [44, 45, 46]. The overexpression of *FAM111B* has also been implicated in hepatocellular carcinoma in a recent study, which highlighted the importance of *FAM111B* in regulating the malignant biological progression of hepatocellular carcinoma through the PI3K/AKT signalling pathway, making it a potential therapeutic target for hepatocellular carcinoma [47].

The Human Molecular Signatures Database pathways (MSigDB) highlighted the Epithelial‐mesenchymal transition pathway as highly related to the GSEA (Figure 7B), which confirms our initial findings documenting the few key pro‐fibrotic markers belonging to categories of growth factor genes involved in signal transduction, inflammatory cytokines and chemokines, as well as epithelial to mesenchymal transition (EMT) genes. This database also annotated the association of cancer and inflammatory pathways, such as the P13K/AKT/mTOR signalling pathway, IL‐6/JAK/STAT3, and notably the TGF‐β. However, the latter was found to be insignificant.

In the context of disease association pathways, the study observed the association of the gene *FAM111B* with rare diseases (Figure 7C,D) using the Rare Diseases AutoRIF ARCHS4 prediction and the Rare Disease GeneRIF ARCHS4 prediction tool. The ARCHS4 web interface was used to explore processed data, which includes average expression across cell lines and tissues, top co‐expressed genes for each gene and predicted biological functions and protein–protein interactions for each gene based on combined existing knowledge with co‐expression. Notably, *FAM111B* did not appear to be directly enriched in these libraries, as only the pro‐fibrotic terms were enriched in these terms. However, the gene‐set library category in diseases reported the input gene library to be enriched in Nephrogenic systemic fibrosis (NFS) and Metachondromatosis (Figure 7D). While *FAM111B* or POIKTMP were not directly highlighted in the rare disease category, its association with NFS is interesting as it mimics the pulmonary fibrosis associated with POIKTMP [48]. Metachondromatosis is a rare genetic cause of osteochondroma and enchondroma production due to a loss of function of the *PTPN11* gene [49]. Although metachondromatosis is not reported in POIKTMP, nor has this condition been associated with FAM111B mutation, certain bone deformities, including osteopenia, have been reported in some cases of POIKTMP [50]. Therefore, the correlation between POIKTMP and metachondromatosis should be further investigated.

## 5 Conclusion

This study provides the first comprehensive analysis of *FAM111B* expression and its association with fibrosis pathways in POIKTMP. We observed the differential expression of FAM111B in specific POIKTMP patient tissues alongside the upregulation of key fibrotic markers. Elucidating the functional consequences of these changes is crucial for understanding how *FAM111B* mutations contribute to POIKTMP pathogenesis and potentially fibrosis in general. Furthermore, a deeper understanding of FAM111B's role in fibrosis could provide important insights into the molecular mechanisms of POIKTMP and other fibrotic diseases, informing future research directions. Hence, *FAM111B* mutations, in conjunction with experimental data generated by cutting‐edge functional genomic technologies, will pave the way for uncovering the functional mechanism of *FAM111B*.

However, further studies are needed to elucidate whether *FAM111B* dysregulation is a primary event or a consequence of the fibrogenic cascade. Our initial focus on upregulated genes provided valuable insights, but downregulated fibrogenic genes may likely contribute to POIKTMP pathology and warrant further investigation. This study represents a significant first step in correlating POIKTMP with the human fibrosis pathway and its association with FAM111B dysregulation. The future exploration of the identified genes and their interactions with the ECM and cell adhesion could provide a deeper understanding of the disease mechanisms and potential therapeutic targets.

Although protein‐level validation is essential to support the transcriptomic findings, the FFPE tissue limited the availability of high‐quality protein in our study, restricting such analyses to a few samples. We acknowledge this limitation and emphasise that future studies utilising fresh or optimally preserved tissue will be crucial for comprehensive proteomic analyses and further validation of our findings. Additionally, the possible role of mutations like those associated with EDS warrants further exploration to understand their contribution to the observed fibrotic phenotype. Lastly, we recognise that another limitation of this study's findings is that it is based on a single patient, which restricts generalizability. Nevertheless, the consistency between the molecular results and the clinical phenotype underscores their value as exploratory data and provides a foundation for future investigations into POIKTMP and other fibrosis‐related disorders.

## Author Contributions

**Nadine Tambwe:** data curation (equal), validation (equal), writing – original draft (equal). **Musalula Sinkala:** formal analysis (equal), visualization (equal), writing – original draft (equal). **Oluwafemi G. Oluwole:** formal analysis (equal), visualization (equal), writing – original draft (equal). **Nonhlanhla P. Khumalo:** conceptualization (equal), funding acquisition (equal), supervision (equal), writing – review and editing (equal). **Afolake Arowolo:** conceptualization (equal), funding acquisition (equal), investigation (equal), project administration (equal), supervision (equal), writing – original draft (equal), writing – review and editing (equal).

## Conflicts of Interest

The authors declare no conflicts of interest.

## Supporting information


**Figure S1:** Validation of the FAM111B gene (c.1861T>G FAM111B p.Y621D) mutation in the extracted DNA from the South African patient with POIKTMP using Sanger sequencing. (A) Skin tissue. (B) Lung tissue. (C) Skeletal muscle tissue. The first two sequence lines in lowercase denote the bi‐allelic FAM111B gene sequences obtained from the control or patient samples used in this study, while the uppercase sequence represents the canonical FAM111B sequence retrieved from NCBI, to which the sequences were aligned. The letters ‘M’, ‘N’ and ‘K’ are standard IUPAC nucleotide ambiguity codes, representing sites with multiple possible nucleotides. In this case, the overlap of T and G nucleotides indicates a heterozygous (i.e., monoallelic) T>G mutation, consistent with the genotype of the South African POIKTMP patient.
**Figure S2:** The disease and pathway enrichment analysis and the GO terms (biological, cellular component and molecular functions) that are significantly associated with the genes of interest in this study.
**Figure S3:** (A) Upregulated gene network of human fibrotic genes associated with FAM11B gene. Relative FAM111B gene expression studies using RT‐qPCR genes associated with FAM111B (Red node). Purple nodes represent selected up regulated fibrotic genes in lung and skin tissue in association with FAM111B. (B) Downregulated fibrotic network in association with FAM111B. Downregulated fibrotic genes in association with FAM111B (Red node). Yellow nodes represent selected down‐regulated fibrotic genes in the lung and skin tissue in association with FAM111B.
**Figure S4:** The protein–protein interaction of FAM111B with the 84 fibrosis pathway proteins. The network properties indicated a highly connected network, with some proteins acting as hubs for multiple interactions.

**Table S1:** PCR (A) and qPCR primer list for mutation validation and FAM111B gene expression (B).
**Table S2:** Primers used for validating some differentially expressed human fibrosis genes from the RT^2^ Profiler PCR Array.

## Data Availability

The data supporting this study's findings are available from the corresponding author upon reasonable request.
