# Supplementary material for: An Integrative Genotyping and Gene Expression Profiling of the Mutated Human FAM111B Gene and Fibrosis‐Associated Pathway in the POIKTMP Syndrome
Source: J Cell Mol Med. 2025 Oct 6;29(19):e70871. doi: 10.1111/jcmm.70871 (PMC12500408; doi:10.1111/jcmm.70871)
Supplement: Supplementary file 1 — Figure S1: Validation of the FAM111B gene (c.1861T>G FAM111B p.Y621D) mutation in the extracted DNA from the South African patient with POIKTMP using Sanger sequencing. (A) Skin tissue. (B) Lung tissue. (C) Skeletal muscle tissue. The first two sequence lines in lowercase denote the bi‐allelic FAM111B gene sequences obtained from the control or patient samples used in this study, while the uppercase sequence represents the canonical FAM111B sequence retrieved from NCBI, to which the sequences were aligned. The letters ‘M’, ‘N’ and ‘K’ are standard IUPAC nucleotide ambiguity codes, representing sites with multiple possible nucleotides. In this case, the overlap of T and G nucleotides indicates a heterozygous (i.e., monoallelic) T>G mutation, consistent with the genotype of the South African POIKTMP patient. Figure S2: The disease and pathway enrichment analysis and the GO terms (biological, cellular component and molecular functions) that are significantly associated with the genes of interest in this study. Figure S3: (A) Upregulated gene network of human fibrotic genes associated with FAM11B gene. Relative FAM111B gene expression studies using RT‐qPCR genes associated with FAM111B (Red node). Purple nodes represent selected up regulated fibrotic genes in lung and skin tissue in association with FAM111B. (B) Downregulated fibrotic network in association with FAM111B. Downregulated fibrotic genes in association with FAM111B (Red node). Yellow nodes represent selected down‐regulated fibrotic genes in the lung and skin tissue in association with FAM111B. Figure S4: The protein–protein interaction of FAM111B with the 84 fibrosis pathway proteins. The network properties indicated a highly connected network, with some proteins acting as hubs for multiple interactions. [file JCMM-29-e70871-s002.docx]

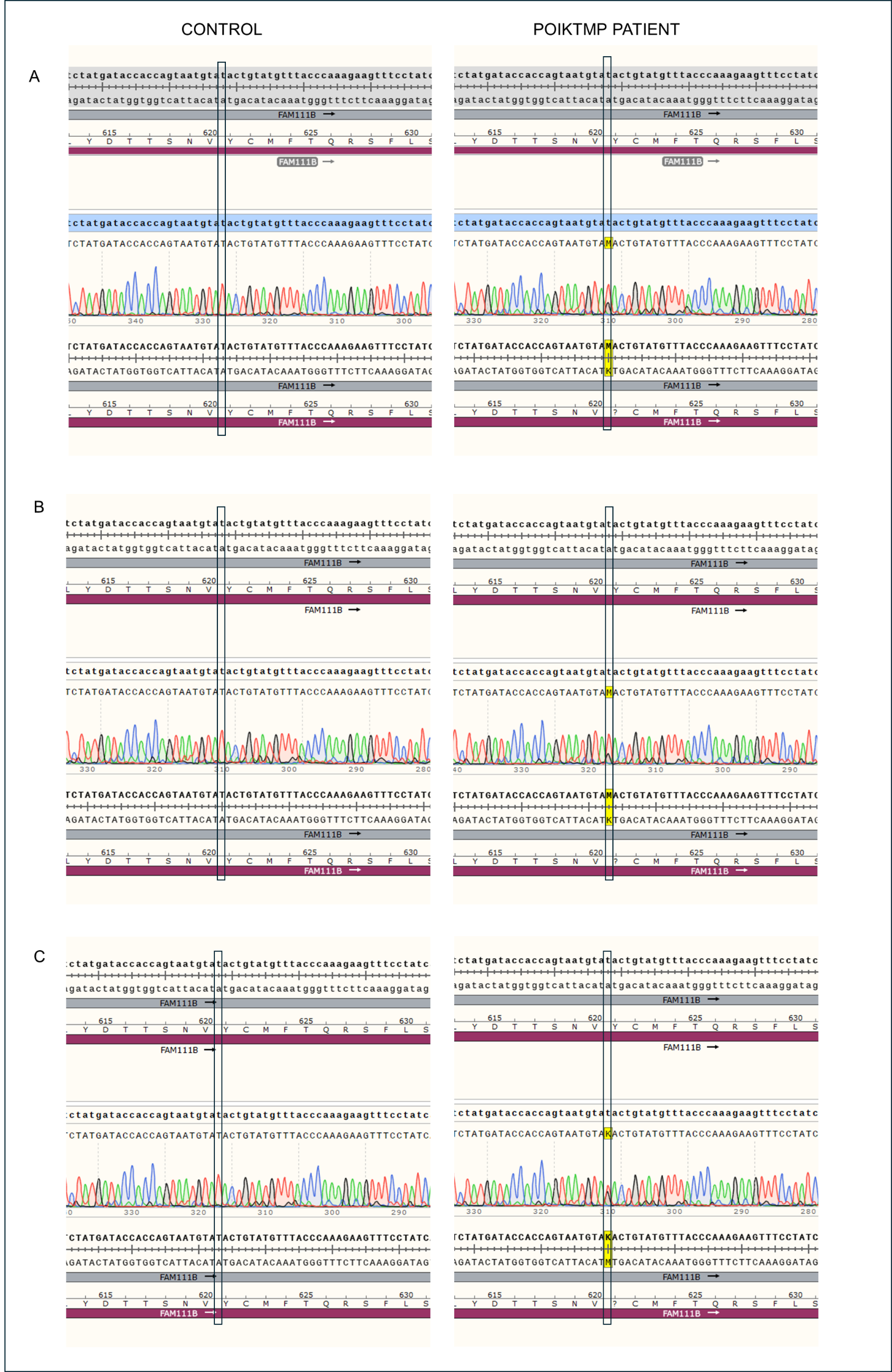


**Supplementary Figure 1:**  Validation of the FAM111B gene (c.1861T>G FAM111B p.Y621D) mutation in the extracted DNA from the South African patient with POIKTMP using Sanger sequencing. (A) Skin tissue. (B) Lung tissue. (C) Skeletal muscle tissue. The first two sequence lines in lowercase denote the bi-allelic FAM111B gene sequences obtained from the control or patient samples used in this study, while the uppercase sequence represents the canonical FAM111B sequence retrieved from NCBI, to which the sequences were aligned. The letters “M,” “N,” and “K” are standard IUPAC nucleotide ambiguity codes, representing sites with multiple possible nucleotides. In this case, the overlap of T and G nucleotides indicates a heterozygous (i.e., monoallelic) T>G mutation, consistent with the genotype of the South African POIKTMP patient.


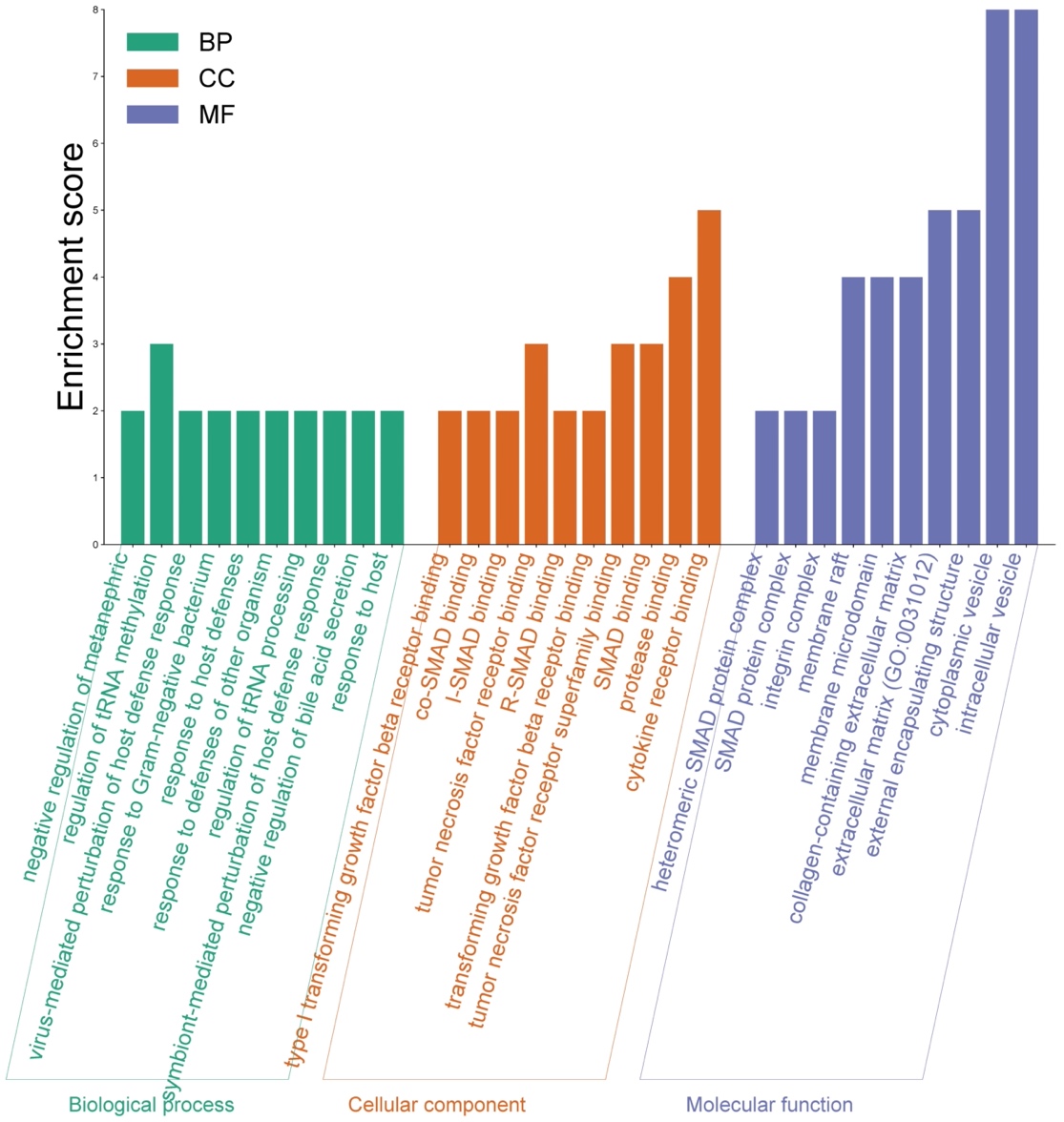


**Supplementary Figure 2**: The disease and pathway enrichment analysis and the GO terms (biological, cellular component and molecular functions) that are significantly associated with the genes of interest in this study.


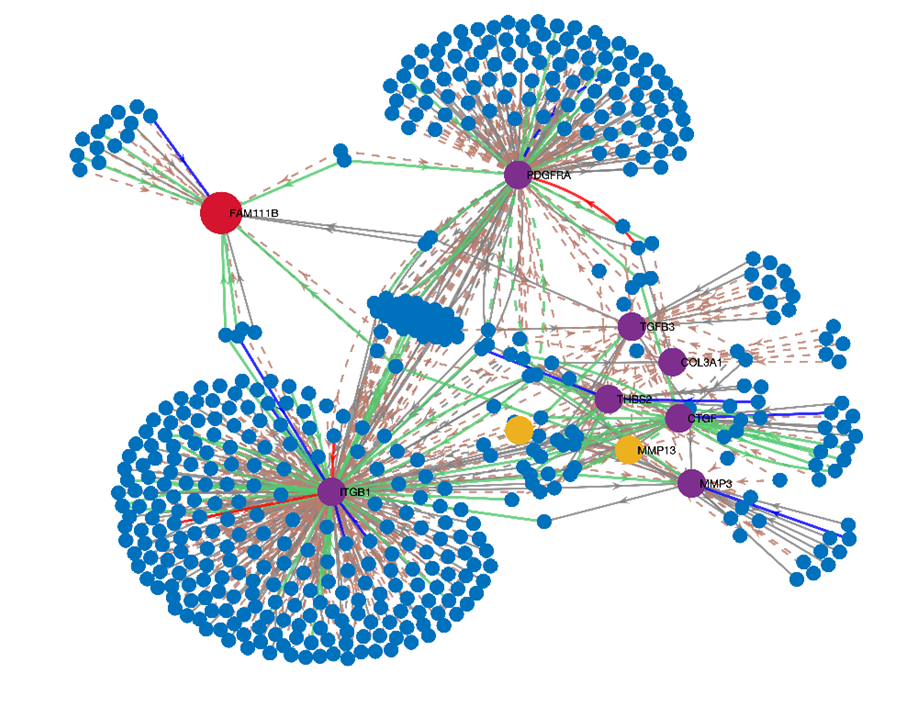


**Supplementary Figure 3a**: Upregulated gene network of human fibrotic genes associated with FAM11B gene. Relative FAM111B gene expression studies using RT-qPCR genes associated with FAM111B (Red node). Purple nodes represent selected up regulated fibrotic genes in lung and skin tissue in association with FAM111B


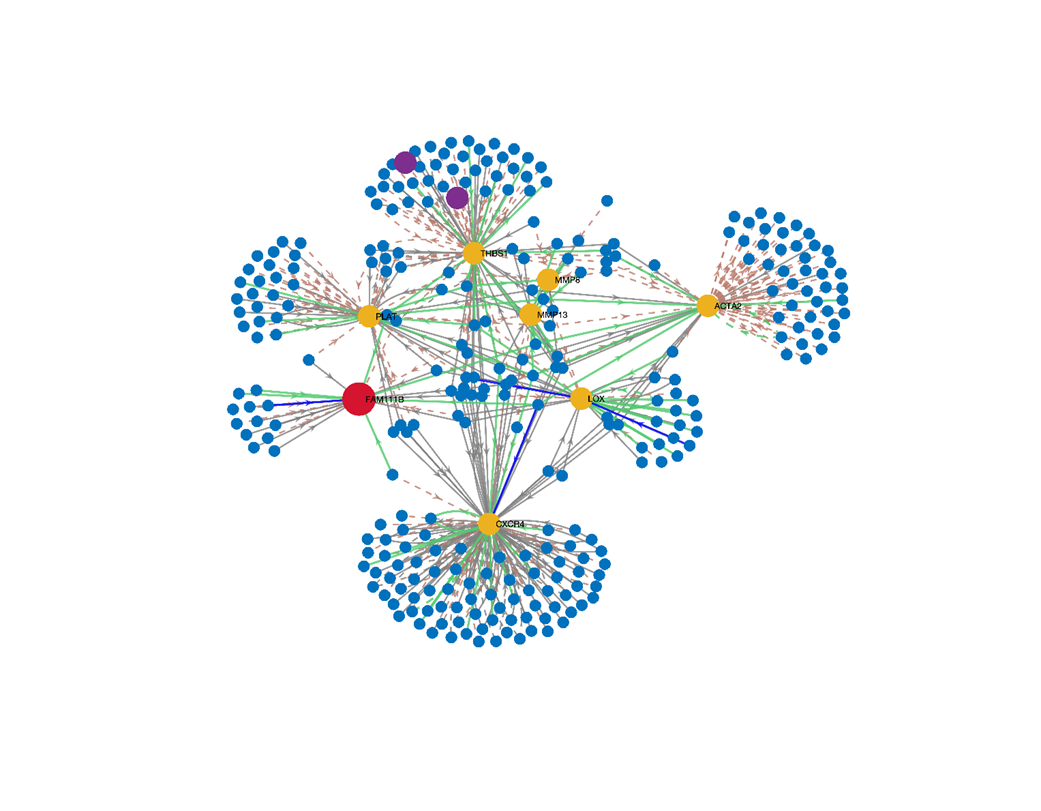


**Supplementary Figure 3b**: Downregulated fibrotic network in association with FAM111B. Downregulated fibrotic genes in association with FAM111B (Red node). Yellow nodes represent selected down-regulated fibrotic genes in the lung and skin tissue in association with FAM111B


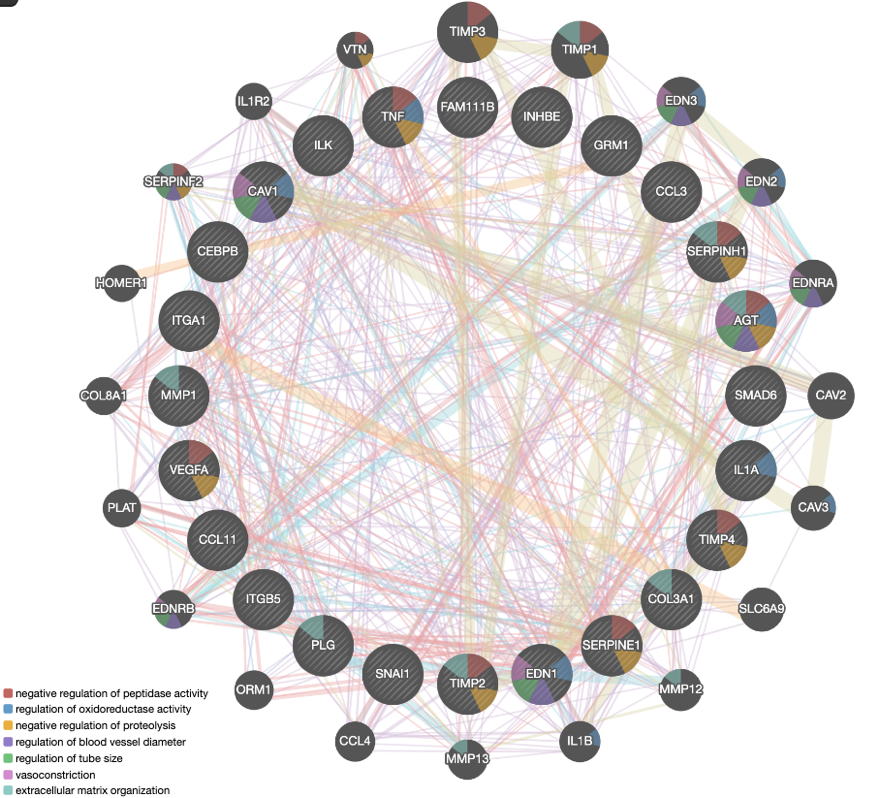


**Supplementary Figure 4**: The protein-protein interaction of FAM111B with the 84 fibrosis pathway proteins. The network properties indicated a highly connected network, with some proteins acting as hubs for multiple interactions.
