# Supplementary material for: An Integrative Genotyping and Gene Expression Profiling of the Mutated Human FAM111B Gene and Fibrosis‐Associated Pathway in the POIKTMP Syndrome
Source: J Cell Mol Med. 2025 Oct 6;29(19):e70871. doi: 10.1111/jcmm.70871 (PMC12500408; doi:10.1111/jcmm.70871)
Supplement: Supplementary file 2 — Table S1: PCR (A) and qPCR primer list for mutation validation and FAM111B gene expression (B). Table S2: Primers used for validating some differentially expressed human fibrosis genes from the RT2 Profiler PCR Array. [file JCMM-29-e70871-s001.docx]

**Supplementary Table 1: PCR (A) and qPCR primer list for mutation validation and FAM111B gene expression (B)**

| **FAM111B (F)** | ATGCTATTAATCTGGATGTCCAAAAGGAGG |
| --- | --- |
| **FAM111B (R)** | CTAACATTCCATGGGTTCAATCTGATGATC |

**(A) PCR (Mutation validation)**

**(B) qPCR Primers:**

| **FAM111B (F)** | GACCGTAGTGTGTTTACAGCA |
| --- | --- |
| **FAM111B (R)** | GGATCCGCACTCCATAGG |
| **GAPDH (F)** | GACCTCAACTACATGGTTTACATG |
| **GAPDH (R)** | GATCTCGCTCCTGGAAGATG |
| **β-ACTIN (F)** | GATTCCTATGGACGAG |
| **β-ACTIN (R)** | GTTGGTGACGGCCGTG |

**Supplementary Table 2: Primers used for validating some differentially expressed human fibrosis genes from the RT2 Profiler PCR Array**

| **Name** | **Forward Sequence** | **Reverse Sequence** |
| --- | --- | --- |
| Platelet-derived growth factor subunit A (PDGFA) | CAGCGACTCCTGGAGATAGAC | GGACAGCTTCCTCGATGCTT |
| Transforming growth factor beta 3 (TGFβ-3) | ATCTGGGAAATGGGCTCGG | TTCTGTCCCCTGCTTCTCTCTC |
| Cellular communication network factor 2 (CCN2) | CACCCGGGTTACCAATGACA | GGATGCACTTTTTGCCCTTCTTA |
| Matrix metallopeptidase 13 (MMP13) | AAGATGCATCCAGGGGTCCT | ATCTCAGGTAGCGCTCTGCAA |
| Matrix metallopeptidase 3 (MMP3) | ACAAAGGATACAACAGGGACCA | GGAACCGAGTCAGGTCTGTG |
| Integrin subunit beta 1 (ITGB1) | TGGTCTCTAAATTGCCGGTGA | AGTGTTGTGGGATTTGCACG |
| Collagen type III alpha 1 chain (COL3A1) | GAAAGAGGATCTGAGGGCTCC | AAACCGCCAGCTTTTTCACC |
| Thrombospondin 2 (THBS2) | TCGGTCCGGAACACTGAAAC | CAGATCTGAGGCTTGCGTG |
